# Isolation, purification and characterization of the antibacterial, antihypertensive and antioxidative properties of the bioactive peptides in the purified and proteolyzed major storage protein of pigeon pea (*Cajanus cajan*) seeds

**DOI:** 10.1016/j.fochms.2021.100062

**Published:** 2021-12-08

**Authors:** Rona Karmela D. Bravo, Mark Rickard N. Angelia, Lawrence Yves C. Uy, Roberta N. Garcia, Mary Ann O. Torio

**Affiliations:** a Institute of Chemistry, Physical Sciences Building, University of the Philippines Los Baños, College Laguna 4031, Philippines; b Institute of Crop Science, College of Agriculture and Food Science, University of the Philippines Los Baños, College, Laguna 4031, Philippines; c Southeast Asian Regional Center for Graduate Study and Research in Agriculture, College, Los Baños, 4031, Laguna, Philippines; d University of the Philippines Los Baños Graduate School, College, Los Baños, 4031, Laguna, Philippines; e German Academic Exchange Service (DAAD), Postfach 200404, D-53134 Bonn, Germany

**Keywords:** Antibacterial, Antihypertensive, Antioxidative, Bioactive peptides, Pigeon pea, *Cajanus cajan*, Globulin

## Abstract

Previous studies in pigeon pea showed health benefits but very few explored peptide bioactivities. This study examined antimicrobial, antioxidant, and antihypertensive activities of peptides in purified and proteolyzed major globulin fraction, cajanin, of pigeon pea (*Cajanus cajan*) seeds. In-silico analysis showed 41 antihypertensive and nine antioxidant peptides but zero antibacterial peptides from cajanin sequence. The Pepsin-Chymotrypsin-Trypsin (PCT) protein digest has no antibacterial activity against *Escherichia coli*, *Candida albicans*, and *Staphylococcus aureus* but has high % ACE inhibition (87.50%). Two HPLC fractions showed low IC_50_ values (HPLC Fraction 1: 0.00535 mg/ml; HPLC Fraction 2: 0.00432 mg/ml), comparable to Captopril (0.00379 mg/ml). This fractions also showed H_2_O_2_ scavenging activity (HPLC Fraction 1: 1.47%; HPLC Fraction 2: 1.51%) and Total Antioxidative Capacity of 0.00088 mg/mL (HPLC Fraction1) and 0.00110 mg/mL (HPLC Fraction 2) ascorbic acid equivalent. Results from this study serve as reference for further investigations of novel pharmaceutical agents that can be derived from legumes.

## Highlights

• Pigeon pea fractions showed high radical scavenging and total antioxidant activity.
• Digested proteins showed comparable ACE-inhibition activity to captopril.
• No antibacterial activity was found in cajanin bioactive peptides.

## 1 Introduction

Dietary proteins have been increasingly acknowledged due to their role as physiologically active compounds. These proteins that are naturally occurring in raw food materials exhibit their biological activity either directly or upon enzymatic hydrolysis in vitro or in vivo. These proteins are known to be a good source of biologically active peptides, or bioactive peptides. Bioactive peptides are specific protein fragments that have a positive impact on body functions or conditions that may influence health. The physiological activity of these peptides can be hindered by the other components of the protein structure, and thus the release of these peptides allows them to manifest their functions. Releasing these peptides from the protein structure can be achieved in three ways: (a) through enzymatic hydrolysis, (b) through hydrolysis by proteolytic microorganisms and (c) through the action of proteolytic enzymes derived from microorganisms or plants (Korhonen and Pihlanto 2006).

Pigeon pea (*Cajanus cajan*) originated in India which developed to have secondary diversity in East Africa. Currently it is grown all over the tropics (Brink 2006). In the Philippines, pigeon pea is grown primarily as a fresh vegetable on a limited scale in the Ilocos Region, Cagayan Valley Region and Batangas. This crop has a great potential for the cropping systems in the country and is a good crop in battling malnutrition as it is a good source of protein (Saxena 2010).

The seeds of pigeon pea are nearly spherical to ovoid, wherein the mature seeds contain 57.3 to 58.7% (fresh weight, fw) carbohydrates, 1.2 to 8.1% crude fiber and 0.6 to 3.8% oil, which is mostly triacylglycerol, the fatty acids (FA's) being linolenic (comprises 5.7% of the total weight), oleic (6.3%) and saturated FA's (37.3%). Vicilin proteins represent approximately 30% of proteins in the mature seeds of pigeon pea, and consist of two major subfractions, cajanin and concajanin (Etonihu et al., 2009).

In this study, the major subfraction of the vicilin protein in pigeon pea seeds, known as cajanin, was isolated, purified and characterized based on the bioactive peptides present in it. The physiological activities of these peptides were determined through antibacterial, antihypertensive and antioxidative activity assays.

## 2 Materials and methods

### 2.1 Plant material

Pigeon peas var. Kadios were purchased from Bureau of Plant Industry-Los Baños Crop Research Development and Production Support Center in October 2011. Protein was immediately extracted and stored in −20˚C.

### 2.2 In silico analysis

The peptide sequences of the major storage protein that exhibits ACE-inhibitory activity and/or antioxidative and/or antibacterial properties were identified from the complete amino acid sequence (Gupta et al., 2006), using the BIOPEP database (http://www.uwm.edu.pl/biochemia), to evaluate the bioactivities exhibited by the major storage protein in pigeon pea seeds.

### 2.3 Protein extraction

#### 2.3.1 Sample preparation

The sample preparation done was based on the method described by Sundaram et al. (1929). The seeds obtained from a local market were washed, sun-dried and powdered into a fine meal using a Wiley® Mill (Thomas Model No. 2) fitted with a 40-mesh sieve.

#### 2.3.2 Crude protein extraction

The crude protein of pigeon pea seeds was extracted using the method described by Sundaram et al. (1929), with slight modifications. About 500 g of the fine meal was treated with 2.5L of 3.5% saline solution. The mixture obtained was stirred vigorously by hand for 20 min. The mixture was then passed through a cheesecloth and was hand-pressed to obtain the water-soluble extract. Any suspended matter from the liquid extract was removed by subjecting the mixture in a centrifuge. The crude extract from the filtered seed meal was obtained and stored in a refrigerator at 4 °C.

#### 2.3.3 Protein content determination

The protein content of Pigeon pea seeds was determined through the Bradford Assay (Bradford 1976), using bovine serum albumin (BSA) as the standard. The absorbance of the mixture was measured at 595 nm using a Shimadzu UV–Visible Spectrophotometer (UVmini-1240).

#### 2.3.4 Ammonium sulfate precipitation

The changes in the level of ammonium sulfate saturation made were 0–40%, 40–60% and 60–90%. The reaction mixture was subjected to centrifugation to obtain the precipitate. Additional amounts of the salt were added into the supernatant liquid to achieve the required degree of saturation. The three precipitates (40%, 60% and 90% fractions) obtained were redissolved in the extraction solution and was dialyzed against distilled water.

#### 2.3.5 Precipitation by dialysis

The redissolved protein was placed in a dialyzing bag and submerged in distilled water with a few drops of 2-mercaptoethanol for 24 h at 4˚C. The different mixtures obtained after dialysis were subjected to centrifugation at 9,184 × g using a refrigerated centrifuge which was set at 4 °C. The precipitate collected was weighed, redissolved in 0.05 M Tris-HCl buffer, pH 8 containing 0.5 M NaCl then stored at 4 °C.

#### 2.3.6 Gel filtration Chromatography (GFC)

The method used for gel filtration chromatography of the dialyzed protein was based on the method of Krishna and Bhatia (1986), with slight modifications. The column was packed with pre-swelled Sephadex G-200 (Sigma® cat. no. G-200–120) in 0.05 M Tris-HCL buffer pH 8 containing 0.4 M NaCl. The protein standard used is a solution containing hemoglobin, bromcresol purple, myoglobin, amyloglucosidase and bovine serum albumin (BSA). Chromatographic separation by gravity alone was performed. A flow rate of 12 ml/hour was done for the elution of the column prior to loading of the sample. The dialyzed 90% ammonium sulfate fraction was selected for further purification and analysis as this was the fraction which showed the approximate molecular weight of the target protein (cajanin, 50 kDa). About 3 ml of fractions were collected. The absorbance of each fraction was monitored at 280 nm and was plotted against elution volume.

### 2.4 Sodium dodecyl sulfate-polyacrylamide gel electrophoresis (SDS-PAGE)

Polyacrylamide gel electrophoresis in the presence of 0.1% sodium dodecyl sulphate (SDS) was carried out in 10% separating gel slabs. The electrophoresis run was conducted at 200 V, followed by staining with 0.5% Coomassie Blue R250. Destaining of the gels was done using two solvent systems: destaining 1 (50% ethanol-10% acetic acid) and 2 (7% ethanol-5% acetic acid). Bio-Rad Precision Plus Protein® Standards (cat. no. 161-0373, 10–250 kDa) was used for the estimation of the molecular weight of the target protein.

A 12-MP digital camera (Nikon Coolpix L22 life series 2010) was used to digitalize the destained SDS-PAGE gels for densitometric analysis using the ImageJ processor application. The software produces plots with peaks that correspond to the color intensity of the bands in the electrophoretogram to be analyzed. The area under each peak was measured and recorded. The degree of digestion, expressed as % remaining protein, was determined by dividing the area of each peak by the area of the peak measured for the undigested sample.

### 2.5 Protein digestion

The digestion mixture contains the digestive enzyme/s and the protein sample in equal concentrations (1 mg/ml). The reaction mixture was allowed to stand for 0, 3, 5, 10, 20, 60 min and 24 h at room temperature. The mixture was then boiled for 5 min to halt the enzymatic hydrolysis. Different enzymes and enzyme combinations were used for the digestion: Pepsin, Chymotrypsin, Trypsin, Pepsin-Trypsin (PT), Chymotrypsin-Trypsin (CT), and Pepsin-Chymotrypsin-Trypsin (PCT). The degree of digestion was assessed through SDS-PAGE analysis.

### 2.6 Preparation of acetone lung powder

The procedure of Folk et al. (1960) was used for the preparation of the lung powder, with slight modifications. Frozen rabbit lungs were used instead of whole swine pancreas. The white, large windpipes were removed to obtain the tissue, which was cut into pieces and then minced in batches using a blender at full speed for 5 min. After the addition of 4 volumes of acetone, the mixture was then homogenized at room temperature using the same blender, at full speed for 6 min. Cheesecloth was then used to remove acetone and soluble components. The residue was then re-suspended in 4 volumes of diethyl ether, followed by homogenizing it at room temperature using a blender at full speed for 6 min. The final dried residue was obtained and spread out onto the cheesecloth for drying at room temperature, under the fume hood. This autolyzed residue was then used for the ACE extraction.

### 2.7 ACE extraction from acetone lung powder

The extraction of ACE from the acetone lung powder was based on the method described by Cushman and Cheung (1971), with some modifications. Ten grams of the rabbit lung powder obtained was homogenized in 100 ml of 50 mM potassium phosphate buffer (pH 8.3) and was placed in a blender at full speed for 1 min. The resulting clear, reddish supernatant was collected, labelled as the crude ACE extract, and stored at 4 °C.

### 2.8 ACE-inhibitory activity assay

The method described by Li et al. (2005) was used for ACE-inhibitory assay, with slight modifications. The ACE-inhibitory activity assay performed is based on the hydrolysis of hippuryl-L-histidyl-L-leucine (HHL) by the enzyme ACE to form Hippuric acid (HA) and histidyl-leucine (HL) as products. For the development of the calibration curve, hippuric acid (HA) is used as the standard. A 0.4 mg/ml stock solution of Hippuric acid (HA) was prepared by dissolving HA powder in 100 mM sodium borate buffer pH 8.3 with 300 mM NaCl. A series of working standard HA solutions were prepared by serial dilution of the stock solution with the same buffer, with concentration range of 0.02 mg to 0.20 mg at 0.02 mg increments. A volume of 500 µL for each concentration of the diluted solutions were mixed with 600 µL of quinoline, placed in a cuvette and mixed using a vortex mixer for 10 s. A volume of 200 µL of benzene sulfonyl chloride (BSC) was added to the mixture then subjected to a vortex mixer for 20 s. About 3.7 ml of ethanol was added to the reaction mixture. The cuvette containing the mixtures were covered with aluminum foil, placed in a 30 °C water bath for 30 min in the dark and were allowed to stand. in in the , T The mixtures were then subjected to colorimetric assay using a UV–Vis spectrophotometer. A calibration curve was obtained by plotting the absorbance at 492 nm versus the HA concentration.

The ACE inhibitor used for this assay include the different protein digest (using different enzyme and enzyme combinations as the sample and using Captopril as the comparator. A volume of 50 µL of 5 mM HHL (in 100 mM sodium borate buffer, pH 8 with 300 mM NaCl) was mixed with 20µLof the ACE inhibitor to produce the reaction mixture. Addition of 10 µL of 100 mU/ml ACE extract followed by incubation at 37 °C warm bath for 30 min was done to initiate the reaction. The reaction was stopped by the addition of 100 µL of 1 M HCl.

The hippuric acid (HA) produced form the reaction was extracted by adding 1.5 ml of ethyl acetate and vortex mixing for 15 s. The removal of ethyl acetate from the organic layer was done by subjecting the mixture to a water bath at 120 °C for 30 min into near dryness. The HA extract was redissolved in 1 ml distilled water and the absorbance was measured at 228 nm using a UV–Vis spectrophotometer. A control was used by mixing all the reagents except for the ACE inhibitor and subjecting the mixture to spectrophotometry.

The inhibitory activity was calculated by:

(1) $$\mathrm{ACE}-inhibitory\;activity\;\left(\%\right)=\frac{B-A}{B-C}\times 100\%$$

where B is the absorbance of the control, C is the absorbance of the reaction blank, and A is the absorbance in the presence the inhibitory protein. The IC_50_ value was determined by regression analysis of fractional ACE-inhibitory activity against log (inhibitor concentration, µM or mg/ml).

### 2.9 High Performance Liquid Chromatography

The protein digest with the lowest molecular weight and the highest % ACE inhibition was identified and submitted to the Analytical Services Laboratory of De La Salle University for High-Performance Liquid Chromatographic analysis. The procedure was done using Agilent Technologies 1200 Series HPLC System equipped with Vydac C18 column (4.6 × 250 mm, 5 µm bead, 30 nm pore size) with a guard column and disposable cartridge (10 nm and 12 µm, respectively). The HPLC fractions that showed the highest peak was collected and was used for the proceeding assays. High Performance Liquid Chromatographic analysis was necessary to separate the major peptide fraction from the rest of the peptides that were all obtained from the digest.

### 2.10 Antimicrobial assay

The method described by Rizzello et al. (2005) was used with slight modifications. A well-diffusion assay was used to measure the antimicrobial assay activity of the protein digest obtained using the 3-enzyme combination Pepsin-Chymotrypsin-Trypsin (PCT) as this was also the digest with the highest % ACE inhibitory activity. *Escherichia coli* 1634 and *Staphylococcus aureus* 1582 on Mueller-Hinton agar (MHA) medium, and *Candida albicans* 2219 on Corn meal agar (CMA) medium were the strains used. All microbes used belong to the National Institute of Molecular Biology and Biotechnology, University of the Philippines - Los Baños, Laguna. Four separate wells using sterile cork borers were made onto each agar plate. A volume of 200 µL each of the diluent, PCT protein digest, distilled water (negative control) and 10% phenol (positive control) were placed onto each well. Each agar plate for each microbial strain were placed in an incubator set at 37 °C for 24 h. The zone of inhibition (in mm) for the sample, the buffer and the controls were measured and compared.

### 2.11 Antioxidative assays

#### 2.11.1 H_2_O_2_-scavenging assay

In this assay, the method used was based on the study of Patel et al. (2010), with n slight modifications. The concentrations of ascorbic acid standard and the samples were adjusted to 0.001 mg/ml. The absorbances of the sample were measured at 230 nm using separate blank samples for reading corrections. The % H_2_O_2_ scavenging activity of each was calculated as:

(2) $$\%\;H_{2}O_{2}\;scavenging\;activity\;=\frac{\left(A_{0}-A_{1}\right)}{A_{0}}\times 100\%$$

where A_0_ is the absorbance of the negative control (contains H_2_O_2_ only) and A_1_ is the absorbance of the sample.

#### 2.11.2 Total antioxidant capacity

The method used in this assay was based on the procedure of Preito et al. (1999), with some modifications. A reagent solution was prepared by combining 0.6 M sulfuric acid, 28 mM sodium phosphate and 4 mM ammonium molybdate. The reducing species used include ascorbic acid (as the standard), the PCT digest and the HPLC fractions. A volume of ascorbic acid stock solution (1 mg/ml) was diluted with distilled water to achieve a final concentration of 0.005 mg/ml. The dilution of the PCT digest and the peptide fractions obtained from HPLC (HPLC fraction 1 and 2) were also done based on the initial concentrations of each (2.26, 0.227 and 0.323 mg/ml respectively) to achieve a final concentration of 0.005 mg/ml. Distilled water was used as the diluent. Each sample were incubated in a hot water bath set at 95 °C for 90 min. After cooling to room temperature, the absorbances of each solution were measured at 695 nm against a blank (containing reagent solution, distilled water and the solvent used for the samples).

A calibration curve was plotted using ascorbic acid as the standard, and the total antioxidant capacity of the samples was expressed as ascorbic acid equivalents via interpolation.

### 2.12 Thin-layer Chromatography

T.he hydrolyzed samples were spotted on a 100 cm × 100 cm TLC plate coated with silica gel, together with the amino acid standards. The developing chamber was pre-equilibrated with 50:30:20 dichloromethane-methanol-acetic acid solvent system. The plates were then placed in the chamber and the solvent was allowed to rise through capillary action until the solvent front is about 1 cm away from the edge of the plate. The plates were removed from the chamber and the solvent front was marked using a pencil. Ninhydrin was used as the visualizing agent. The plates were placed on an iodine chamber to clearly visualize the faint spots. Two replications for each HPLC fraction were run and the average Rf from the two trials for each of the HPLC fractions 1 and 2 were used to determine possible amino acid sequences.

The retardation factor, Rf, of each fraction was calculated using the equation:

(3) $$\mathrm{Rf}=\frac{\mathrm{distance}\;traveled\;by\;sample}{\mathrm{distance}\;traveled\;by\;solvenet}$$

The Rf values of each fraction was compared to that of the amino acid standards to determine the amino acid composition of the fractions (Boyer 1993).

### 2.13 Statistical analysis

All samples were analyzed in three replications. One-way analysis of variance (ANOVA) assuming equal (Welch’s) and unequal variances (Fisher’s) were both used to test the significance of differences (P < 0.05) between conditions. Additional stat analyses such as Homogeneity of variances test (Levene’s), Normality Test (Shapiro-Wilk), and post-hoc test was done. Post-hoc analysis Games-Howell Post-Hoc test for unequal variances was performed to see significant differences between means. All statistical analyses were performed using jamovi (The jamovi project, 2021a, The jamovi project, 2021b).

## 3 Results

### 3.1 In silico analysis

The BIOPEP database (http://www.uwm.edu.pl/biochemia) was used as reference to determine the following amino acid sequences exhibiting the specified bioactivity. Amino acid sequences exhibiting antibacterial activity were not found in the complete amino acid sequence of the target protein (Table 1).

**Table 1:** Bioactive peptides that exhibit antihypertensive and antioxidative activities that are found in the sequence of cajanin from Pigeon peas.

Peptide sequence	Location	Activity
TK	[3–4]	ACE inhibition
VF	[9–10]	ACE inhibition
VP	[17–18]	ACE inhibition
KYL	[19–21]	ACE inhibition
IY	[26–27]	ACE inhibition
EYK	[31–33]	ACE inhibition
KPR	[35–37]	ACE inhibition
LFLPQY	[39–44]	ACE inhibition
DA	[46–47]	ACE inhibition
ILVV	[50–53]	ACE inhibition
SGKA	[55–58]	ACE inhibition
SF	[70–71, 115–116, 152–153, 231–231]	ACE inhibition
LVRGDT	[55–58]	ACE inhibition
KL	[80–81]	ACE inhibition
AGTIAYLA	[83–90]	ACE inhibition
VLDL	[100–103]	ACE inhibition
YNKPGQLQS	[107–115]	ACE inhibition
SGTQ	[119–122]	ACE inhibition
SGF	[129–131]	ACE inhibition
EAGCF	[137–141]	ACE inhibition
GD	[75–76, 147–148, 240–241, 280–281]	ACE inhibition
KGSFR	[150–154]	ACE inhibition
TGTRA	[156–160]	ACE inhibition
TQK	[162–164]	ACE inhibition
RR	[169–170]	ACE inhibition
EI	[172–173]	ACE inhibition
IE	[185–186]	ACE inhibition
PVPK	[194–197]	ACE inhibition
SGT	[119–121, 203–205]	ACE inhibition
TGS	[216–218]	ACE inhibition
RF	[227–228]	ACE inhibition
LYIGD	[237–241]	ACE inhibition
GQ	[111–112,246–247]	ACE inhibition
YKE	[256–258]	ACE inhibition
VR	[74–75, 273–274, 317–318]	ACE inhibition
KLPGDVFVIPAGHP	[276–290]	ACE inhibition
AI	[292–293]	ACE inhibition
LN	[299–300]	ACE inhibition
IGFGILA	[302–308]	ACE inhibition
NY	[310–311]	ACE inhibition
SLVR	[315–318]	ACE inhibition
LK	[21–22, 63–64]	Antioxidation
IY	[26–27,213–214]	Antioxidation
KP	[35–36,109–110]	Antioxidation
IKLPAG	[79–84]	Antioxidation
AY	[87–88]	Antioxidation
KD	[167–168]	Antioxidation
EL	[187–188, 243–244]	Antioxidation
IY	[26–27,213–214]	Antioxidation
PAG	[82–84,286–288]	Antioxidation

Source: Minkiewicz P., Dziuba J., Iwaniak A., Dziuba M., Darewicz M., BIOPEP database and other programs for processing bioactive peptide sequences. Journal of AOAC International, 91, 2008, 965–980.

### 3.2 Protein digestion

**Fig. 1:**
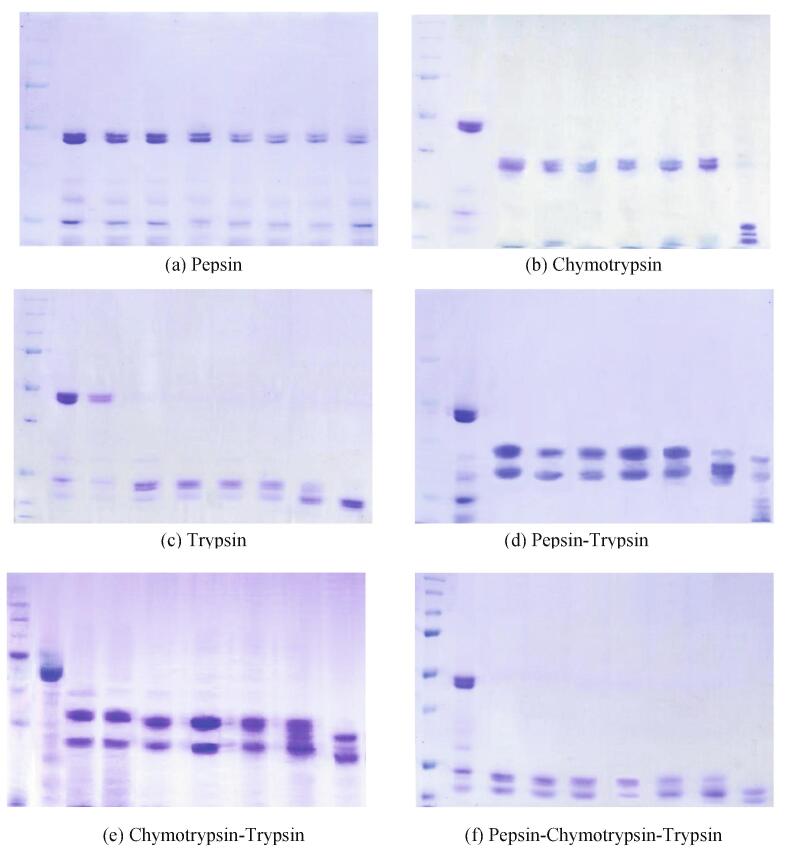
SDS-PAGE profile of the protein digests using different enzymes and enzyme combinations. **Lane 1:** Protein MW standard: 10–225 kDa**; Lane 2:** Undigested purified protein**; Lanes 3 to 9:** 0, 3, 5, 10, 20, 60 mins, 24 h-digests, respectively.

The purified protein was digested using different enzymes: Trypsin, Chymotrypsin and Pepsin, and combinations of each: Chymotrypsin-Trypsin, Pepsin-Trypsin and Pepsin-Chymotrypsin-Trypsin. The SDS-PAGE profile for each type of enzyme and enzyme combination was analyzed and compared to one another (Fig. 1).

Trypsin showed a more effective hydrolysis of the globulin in comparison with Pepsin and Chymotrypsin, even at 0-minute digestion since it showed several distinct bands, with molecular weights less than 25 kDa.

Different combinations of these enzymes were also used in the digestion of the globulin. The efficiency of each enzyme combination was evaluated based on their electrophoretograms.

Most of the SDS-PAGE profiles shows that greater better denaturation at longer digestion periods. However, the enzyme combination Pepsin-Chymotrypsin-Trypsin (PCT) showed a more efficient digestion, even at 0-minute digestion period. Thus, the digests obtained from the digestion of the purified globulin using the combination of the three enzymes were used for the HPLC analysis and other proceeding assays.

### 3.3 Densitometric analysis

Different peaks were obtained corresponding to the color intensity of each band seen in each of the SDS-PAGE profile (Appendix 3). The peak representing the undigested protein on SDS-PAGE is expected to have the largest area. There was a subsequent decrease in the area under each peak as the time of digestion increased, regardless of enzymes and enzyme combinations. The degree of digestion increased as the time of digestion increased, as represented by the decrease in the % remaining protein calculated for each enzyme and enzyme combination used. In contrast with the SDS-PAGE profile, the densitometric analysis showed that the enzyme chymotrypsin had the most effective digestion. However, it can be observed from the SDS-PAGE profile that the digests obtained from these two (Chymotrypsin alone and Triple enzyme combination) had different molecular weight, i.e., the digest obtained from the triple enzyme combination produced a digest that has a lower molecular weight.

### 3.4 High Performance Liquid Chromatographic analysis

**Fig. 2:**
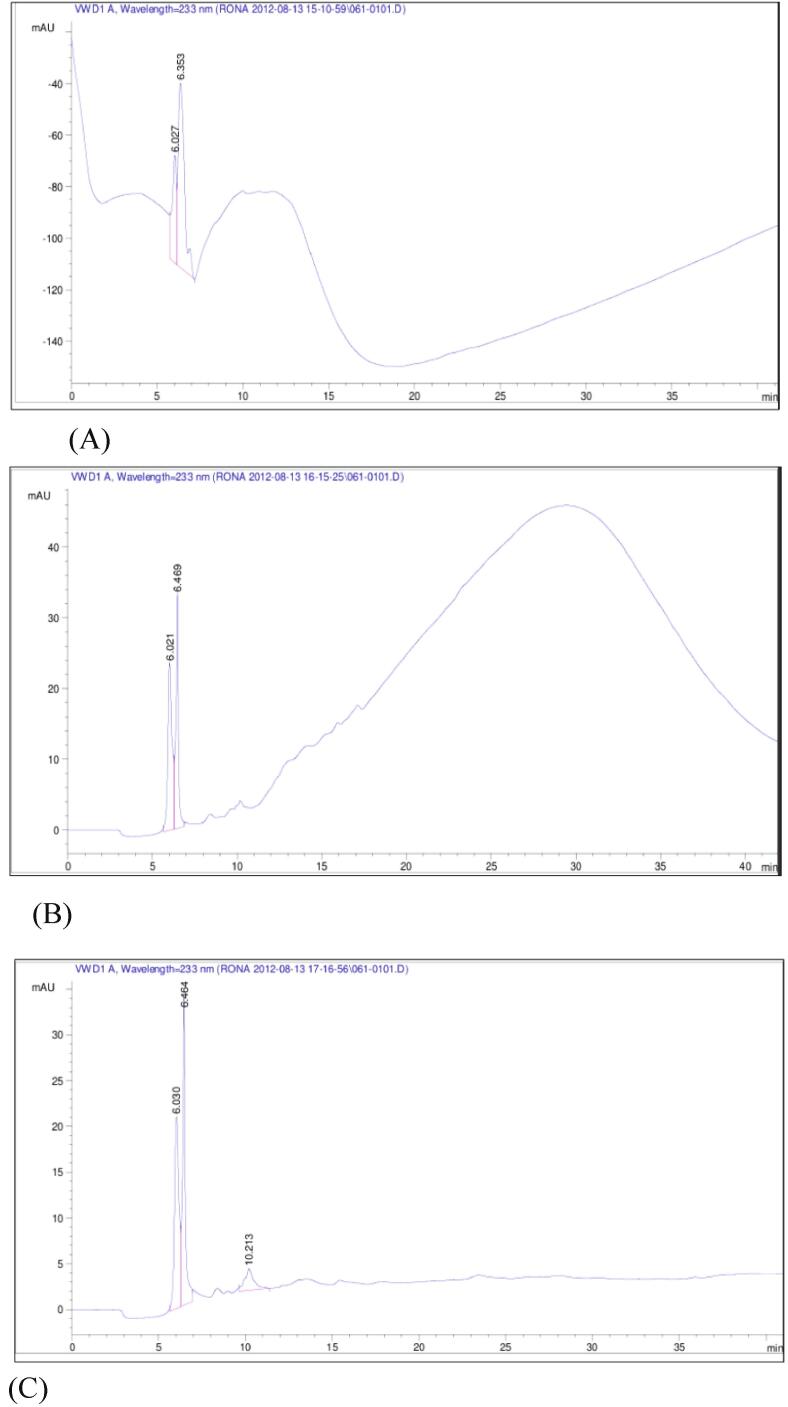
The elution profiles obtained from each trial (A-Trial 1, B-Trial2, C-Trial3) after subjecting the three-enzyme digest (PCT) in High Performance Liquid Chromatography. Eluents obtained at an average elution time of 6 min.

Three trials were performed for HPLC analysis, The protein digest with the lowest molecular weight and the highest % ACE inhibition was identified and submitted to the Analytical Services Laboratory of De La Salle University for High-Performance Liquid Chromatographic analysis. The procedure was done using Agilent Technologies 1200 Series HPLC System equipped with Vydac C18 column (4.6 × 250 mm, 5 um bead, 30 nm pore size) with a guard column and disposable cartridge (10 nm and12 um respectively). The HPLC fractions that showed the highest peak was collected and was used for the proceeding assays. For each trial there were two different fractions that were collected; with elution times that are within the range of 6.021–6.469 min. Elution profiles obtained are shown in Fig. 2. Eluents were collected and stored at 4 °C.

### 3.5 Antibacterial activity assay

There was no significant zone of inhibition showed by the PCT digest for the three microbial strains, only the positive control (10% phenol) showed positive results (Appendix 4). Thus, it can be concluded that the globulin protein has no antimicrobial activity against these strains. This observation confirmed the results of the in-silico analysis where no amino acid residue of cajanin was found to have antibacterial activity (Table 1).

### 3.6 Antioxidative activity assay

#### 3.6.1 Hydrogen peroxide scavenging activity assay

**Fig. 3:**
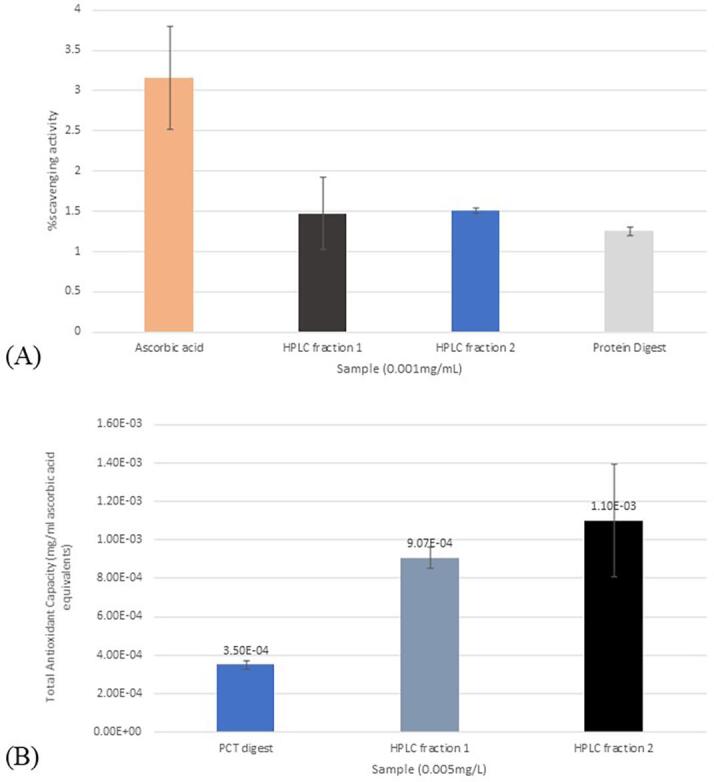
Antioxidant activity of PCT digest and fractions; **A**·H_2_O_2_-scavenging activity of PCT digest and fractions compared to ascorbic acid; **B.** Total antioxidant activity of PCT digest and fractions expressed in mg/ml ascorbic acid equivalents.

Preliminary analysis was done to assess the H_2_O_2_-scavenging activity of the two HPLC fractions in comparison with the PCT digest and ascorbic acid. Different volumes of each sample were obtained to achieve a final concentration of 0.001 mg/ml. From the graph shown in Fig. 3, ascorbic acid showed the highest the H_2_O_2_-scavenging activity (3.16%), followed by the two fractions (Fraction 1: 1.47%, Fraction 2: 1.51%). The PCT digest showed the lowest H_2_O_2_-scavenging activity, having a value of 1.25%. Both Welch’s ANOVA for unequal variances (p-value (0.023) < level of significance (0.05)) and Fischer’s ANOVA for equal variances (p-value (0.004) < level of significance (0.05)) showed that there is at least one significantly different mean. Based on the Normality test (Shapiro-Wilk Test), the data follows normal distribution (p-value (0.324) > level of significane (0.05)). Test for homogeneity of variances (Levene’s test) was also done. Since p-value (0.016) for Levene’s test is less than the level of significance (0.05), there is enough evidence to conclude that the data does not have homogenous variances thus Games-Howell Post-Hoc test for unequal variances was used. Based on the Post-Hoc test done, there is no significant differences between means of ascorbic acid, HPLC fraction1 and 2, and the protein digest. This suggests that the H_2_O_2_-scavenging activity of the HPLC fractions and the protein digest is comparable. However, there is a significant difference in the means of HPLC fraction 2 and the protein digest which indicates significantly higher H_2_O_2_-scavenging activity of HPLC fraction 2 compared to the protein digest (Appendix 5) (Fox and Weisberg, 2020, R Core Team, 2020, The jamovi project, 2021a).

#### 3.6.2 Total antioxidant capacity

The results show that, at a given concentration, the two fractions, HPLC Fraction 1 and HPLC fraction 2, have higher total antioxidant capacities (0.00088 mg/ml and 0.00110 mg/ml ascorbic acid equivalents, respectively) than the PCT digest (0.00035 mg/ml ascorbic acid equivalents), and that HPLC Fraction 2 showed a higher value than HPLC Fraction 1 (Fig. 3). Both Welch’s ANOVA for unequal variances (p-value (0.002) < level of significance (0.05)) and Fischer’s ANOVA for equal variances (p-value (0.012) < level of significance (0.012)) showed that there is at least one significantly different mean. The Levene’s test for homogeneity of variances and Shapiro-Wilk test for normality were done to check if the assumptions in doing Fisher’s ANOVA and Tukey’s post-hoc test is satisfied. The data follows normal distribution (p-value (0.151) > level of significance (0.05)) but the variances are not homogenous (p-value (0.029) < level of significance (0.05)), thus Games-Howell Post-Hoc test for unequal variances was used. There are no significant differences except between the means of HPLC fraction 1 and the PCT digest. This means that the Total Antioxidant Activity of HPLC Fraction 1 is significantly higher than the PCT digest (Appendix 6) (Fox and Weisberg, 2020, R Core Team, 2020, The jamovi project, 2021a).

### 3.7 ACE-inhibitory activity assay

The 24-hour protein digests obtained using different enzymes and combinations of each were subjected to ACE assay to compare and relate the % inhibition calculated for each.

The protein digest obtained using the combination Pepsin-Chymotrypsin-Trypsin (PCT) showed the highest % ACE inhibition among other digests. Thus, this digest was used for further analysis. Both Welch’s ANOVA for unequal variances (p-value (<0.001) < level of significane (0.05)) and Fischer’s ANOVA (p-value (<0.001) < level of significane (0.05)) for equal variances shows that there is at least one significantly different mean. The Levene’s test for homogeneity of variances and Shapiro-Wilk test for normality were done to check if the assumptions in doing Fisher’s ANOVA and Tukey’s post-hoc test is satisfied. The data follows normal distribution (p-value (0.127) > level of significance (0.05)) but the variances are not homogenous (p-value (0.004) < level of significance (0.05)), thus Games-Howell Post-Hoc test for unequal variances was used. From the analysis, there is enough evidence to conclude that there is a significant difference in between the means of Pepsin and PCT (Pepsin-Chymotrypsin-Trypsin), Pepsin and PT(Pepsin-Trypsin), and PCT and PT. (Appendix 7) (Fox and Weisberg, 2020, R Core Team, 2020, The jamovi project, 2021a).

**Fig. 4:**
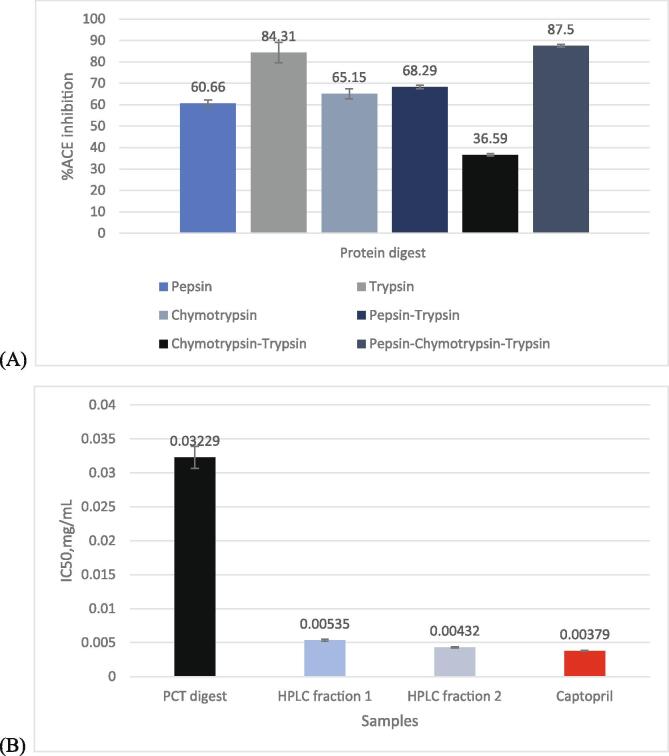
ACE inhibition activity of protein digests and fractions against positive control (Captopril): **A.** ACE inhibition activity (%) of different protein digests; **B.** IC_50_ values of the protein digests and the HPLC fractions.

The IC_50_ value of the PCT digest and the HPLC fractions were determined through ACE assay, with Captopril as the reference. The HPLC fractions showed lower IC_50_ values (Fraction 1: 0.00535 mg/ml; Fraction 2: 0.00432 mg/ml) than the PCT digest (0.03229 mg/ml) but are comparable to that of Captopril (0.00379 mg/ml). Furthermore, HPLC Fraction 2 showed lower IC_50_ value than Fraction 2 (Fig. 4). Both Welch’s ANOVA for unequal variances (p-value (<0.01) < level of significance (0.05)) and Fischer’s ANOVA for equal variances (p-value (<0.01) < level of significance (0.05)) shows that there is at least one significantly different mean. Based on the Shapiro-Wilk test for normality, the data follows normal distribution (p-value (<0.002) < level of significance (0.05)) but the variances of the data is not homogenous based on Levene’s test for homogeneity of variances (p-value (0.001))thus Games-Howell Post-Hoc test for unequal variances was used. There is a significant difference between all the means including captopril against HPLC fraction1,2, and PCT digest (Appendix 7) (Fox and Weisberg, 2020, R Core Team, 2020, The jamovi project, 2021a).

### 3.8 Thin-Layer Chromatography

The two HPLC fractions were subjected to Thin-Layer Chromatography to determine their amino acid composition and deduce their possible amino acid sequences.

The Rf values obtained for the two fractions were then compared to that of the standards to determine their possible amino acid sequence based on the in silico analysis (Table 2). The possible amino acid sequences for Fraction 1 include RA, RR and DA, while for Fraction 2, the deduced amino acid sequences were LK, KL, RA, RR and DA. The in-silico analysis showed that these sequences were found to exhibit antioxidative and antihypertensive activities (Table 1).

**Table 2:** The amino acid composition and the possible amino acid sequence of the two HPLC fractions.

Fraction	Possible Amino Acid Sequence	Reference
Fraction 1	RA	Cushman et al., 1981
	RR	Sentandeu and Toldra, 2007
	DA	Cushman et al., 1981
Fraction 2	LK	Huang et al., 2010
	KL	Meisel et al., 2006
	RA	Cushman et al., 1981
	RR	Sentandeu and Toldra, 2007
	DA	Cushman et al., 1981

## 4 Discussion

### 4.1 Antibacterial assay

Although there is no antibacterial activity observed against C. albicans, E. coli, and S. aureus, this does not disprove that some legumes have antibacterial properties as previously stated in different references (Kanatt et al., 2011, Pina-Pérez and Pérez, 2018, Hori et al., 2006, El-Araby et al., 2020).

### 4.2 Total antioxidant capacity

Of the two fractions, Fraction 2 have the higher reducing power than Fraction 1 (22.2% difference), but both were greater than PCT digest.

This assay measures the reducing power of the samples, expressed in ascorbic acid equivalents, which can be considered as a reflection of antioxidant activity (Oktay et al., 2003). Having high values for total antioxidant capacity would mean that Fractions 1 and 2 have high reducing power. These results can be used to assess their ability as primary and secondary antioxidants, in terms of electron-donating properties and capacity to reduce the oxidized intermediates of lipid peroxidation (Yen and Chen, 1995).

### 4.3 Hydrogen peroxide scavenging activity assay

The results imply that the isolated peptides were more effective in scavenging H_2_O_2_, in comparison with the PCT digest, and thus may inhibit hydroxyl radicals (OH·) to cause lipid peroxidation and DNA damage (Chanda and Dave, 2009). This also implies that the purification and digestion were necessary to produce peptides that exhibit radical-scavenging activities.

In a different study, the globulin fraction from rice bran protein, as well as its peptic and tryptic hydrolysates, was found to have the highest antioxidative activity in terms of its %inhibition of lipid peroxidation, in comparison with the prolamin, albumin and glutelin fraction. In addition, the enzymatic hydrolysates were greater in antioxidative activity than those of their corresponding proteins before digestion. This can be attributed by the resultant peptides and/or amino acids which were produced from the digestion of the proteins (Chanput et al., 2009).

### 4.4 ACE-inhibitory activity assay

The potency of an inhibitor is usually not based on the inhibition percentage, since this value is based on a single inhibitor concentration only. A concentration–response plot of % inhibition (or fractional activity) as a function of inhibitor concentration is usually generated, where the potency of the inhibitor is assessed by determining the midpoint value in the plot. This midpoint value is known as the IC_50_ value, which is equal to the concentration of the inhibitor which results to a 50% reduction of the target enzyme activity (Copeland, 2005), which in this study, is the activity of Angiotensin-converting enzyme (ACE). Thus, a lower IC_50_ value would mean a greater inhibitor potency since an inhibitor with a low IC_50_ value reduces the ACE activity by 50% even at low concentrations.

In the comparison of the IC_50_ values of the PCT digest and the HPLC fractions, it can be stated that the HPLC fractions have greater ACE-inhibitor potency than PCT and thus have greater antihypertensive activities. In addition, comparison of the IC_50_ values for the two fractions with that of captopril shows that the values obtained are comparable having a % difference of 34.1% for Fraction 1 and 13.1% for Fraction 2.

Captopril was chosen as reference since it is known as a potent, competitive inhibitor of ACE and is thus known to be used in the treatment of hypertension. Its IUPAC name is (2S)-1-[(2S)-2-methyl-3-sulfanylpropanoyl] pyrrolidine-2-carboxylic acid, having the molecular formula C_19_H_15_NO_3_S. Its mechanism of action lies in the fact that it competes with Angiotensin I for binding to ACE and inhibits the enzymatic proteolysis of Angiotensin I to Angiotensin II. Thus, captopril helps in decreasing the concentration of Angiotensin II, which is an octapeptide responsible in increasing the blood pressure and blood volume (Campbell et al., 2008).

## 5 Conclusions

The results showed that peptides from pigeon pea have antioxidant and anti-hypertensive activities but has no antibacterial properties. PCT digest has up to 87.50% ACE inhibition while two fractions have IC_50_ values comparable to captopril. This fractions also showed H_2_O_2_ scavenging activity (Fraction 1: 1.47%; Fraction 2: 1.51%) and Total Antioxidative Capacity of 0.00088 mg/mL and 0.00110 mg/mL ascorbic acid equivalent for fractions 1 and 2, respectively.

Overall, this shows that there are a lot of potential for pigeon pea. Studying its bioactive peptides and even other metabolites can be useful in development of food supplements and even in alternative medicine.

## Author contributions

Ms. Rona Karmela D. Bravo worked on the isolation, purification, and characterization of the major storage protein as well as that of the bioactive peptides found in pigeon pea seeds. Dr. Mary Ann O. Torio served as Ms. Bravo's adviser for her laboratory work while both Dr. Mary Ann O. Torio and Mr. Lawrence Yves C. Uy were responsible for editing the manuscript. Mr. Lawrence Yves C. Uy was also responsible for the graphical representations, statistical analyses, and image improvement**.** Dr. Rickard N. Angelia and Dr. Roberta N. Garcia served as Ms. Bravo's thesis panel members and have always been available for consultation and guidance.

## Declaration of Competing Interest

The authors declare that they have no known competing financial interests or personal relationships that could have appeared to influence the work reported in this paper.
