# Supplementary material for: Isolation, purification and characterization of the antibacterial, antihypertensive and antioxidative properties of the bioactive peptides in the purified and proteolyzed major storage protein of pigeon pea (Cajanus cajan) seeds
Source: Food Chem (Oxf). 2021 Dec 8;4:100062. doi: 10.1016/j.fochms.2021.100062 (PMC8991556; doi:10.1016/j.fochms.2021.100062)
Supplement: Supplementary data 1 [file mmc1.docx]

Appendix


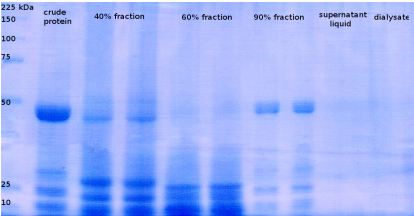


Appendix 1. SDS-PAGE profile showing the degree of each purification step (ammonium sulfate fractionation).


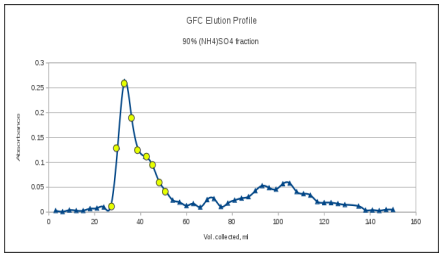


(A)


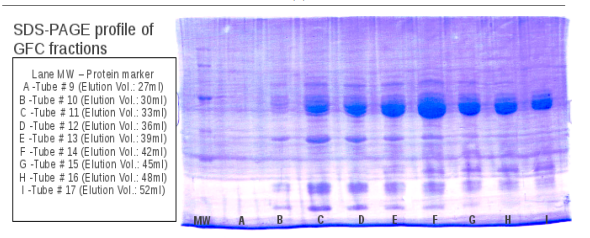


(B)

Appendix 2. Gel Filtration Chromatography: (a) the GFC elution profile obtained for the 90% ammonium sulfate fraction; (b) the SDS-PAGE profile of the selected fractions.


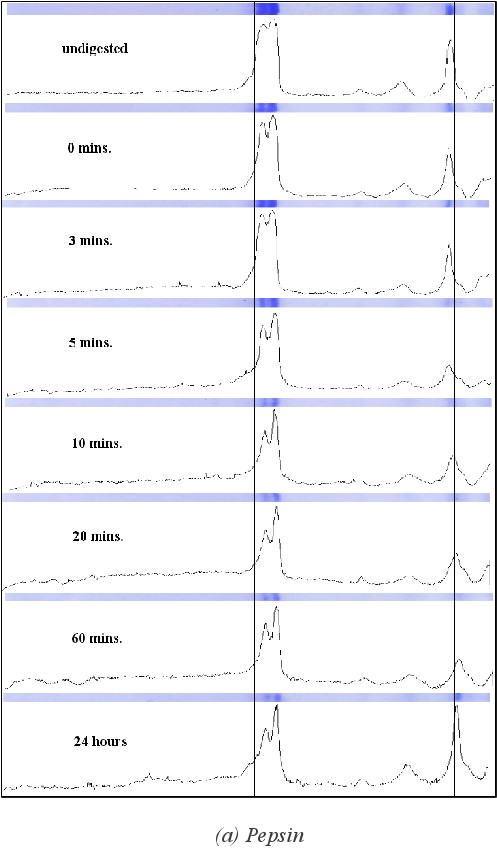


1. Pepsin


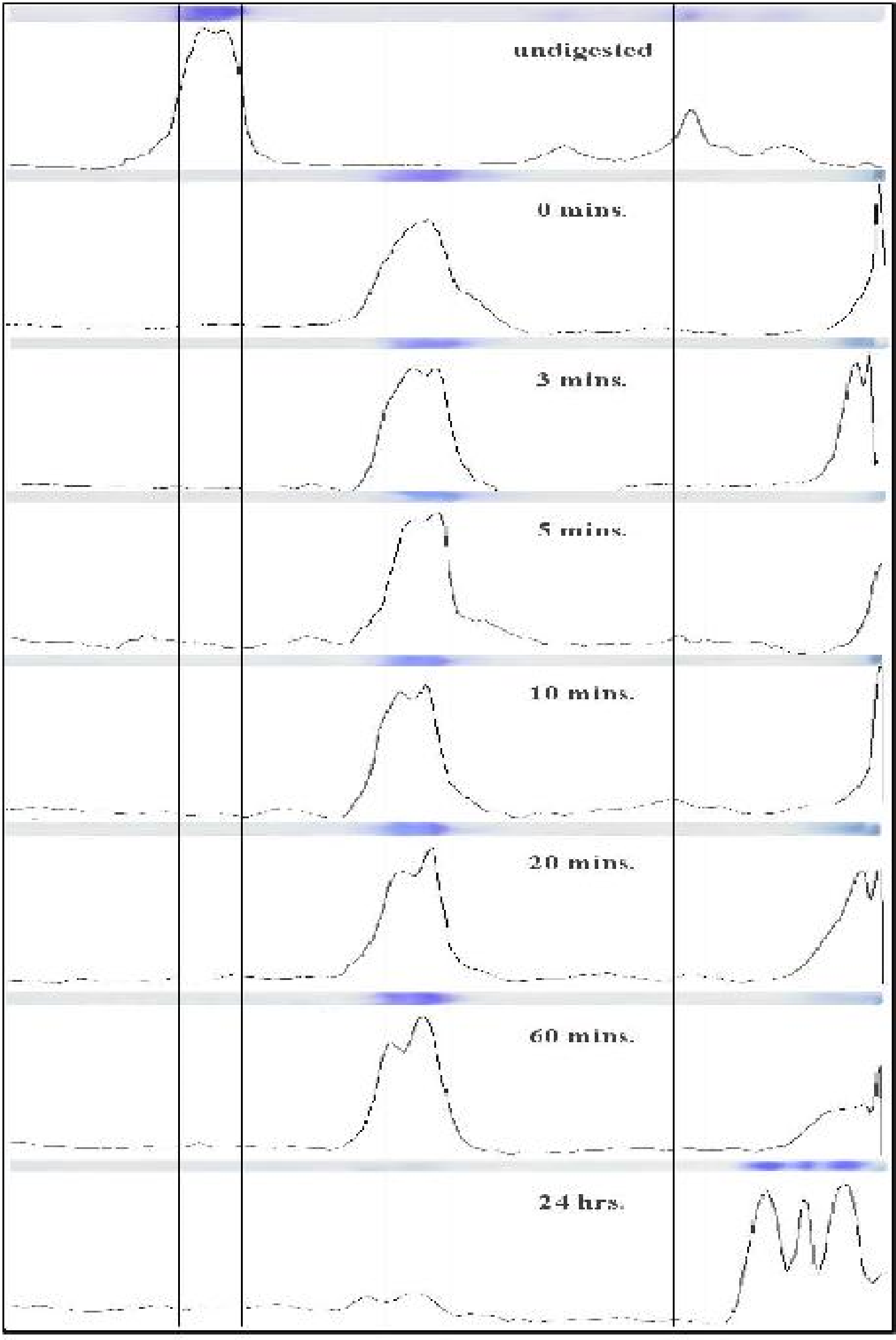


(b) Chymotrypsin

1. Chymotrypsin


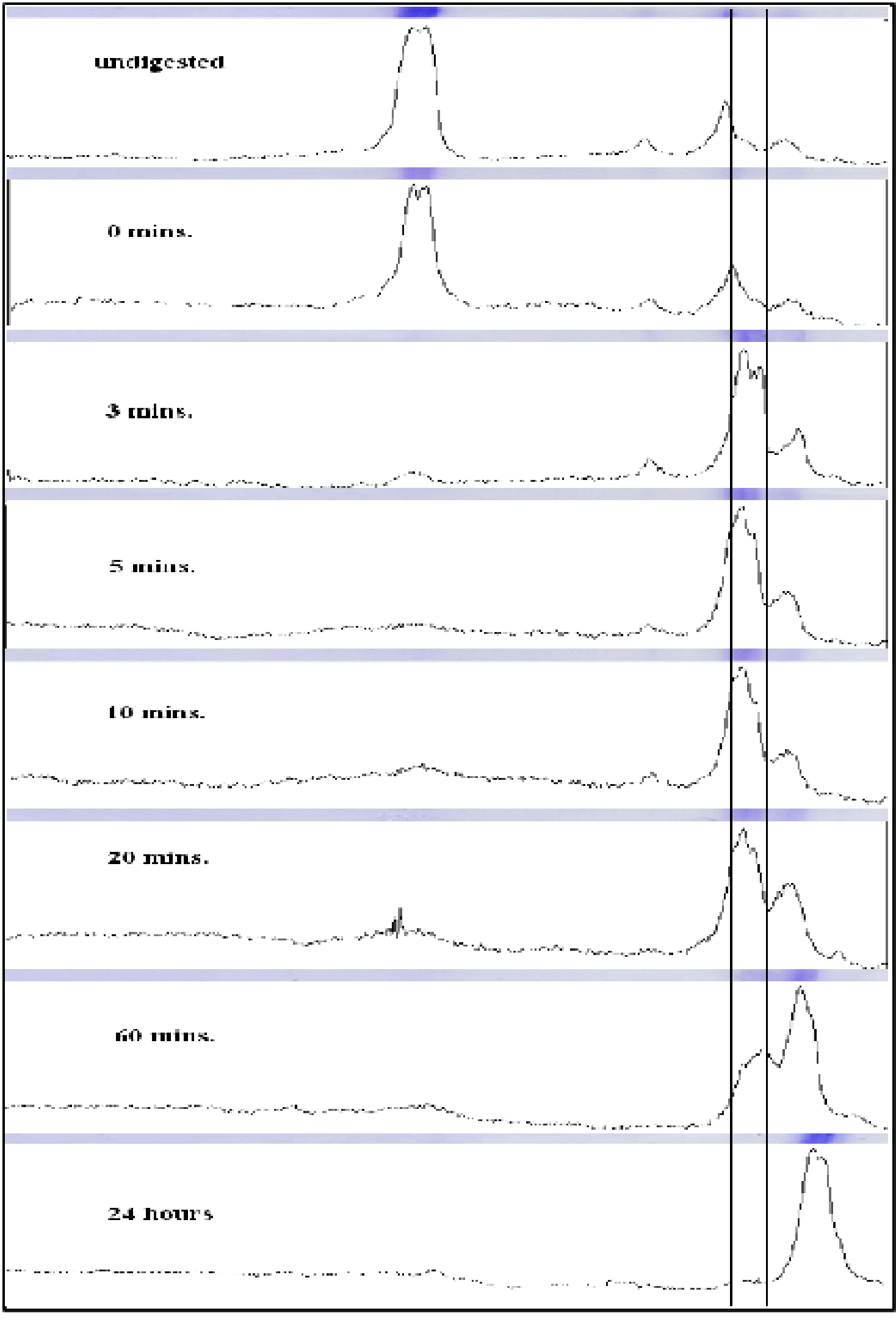


1. Trypsin


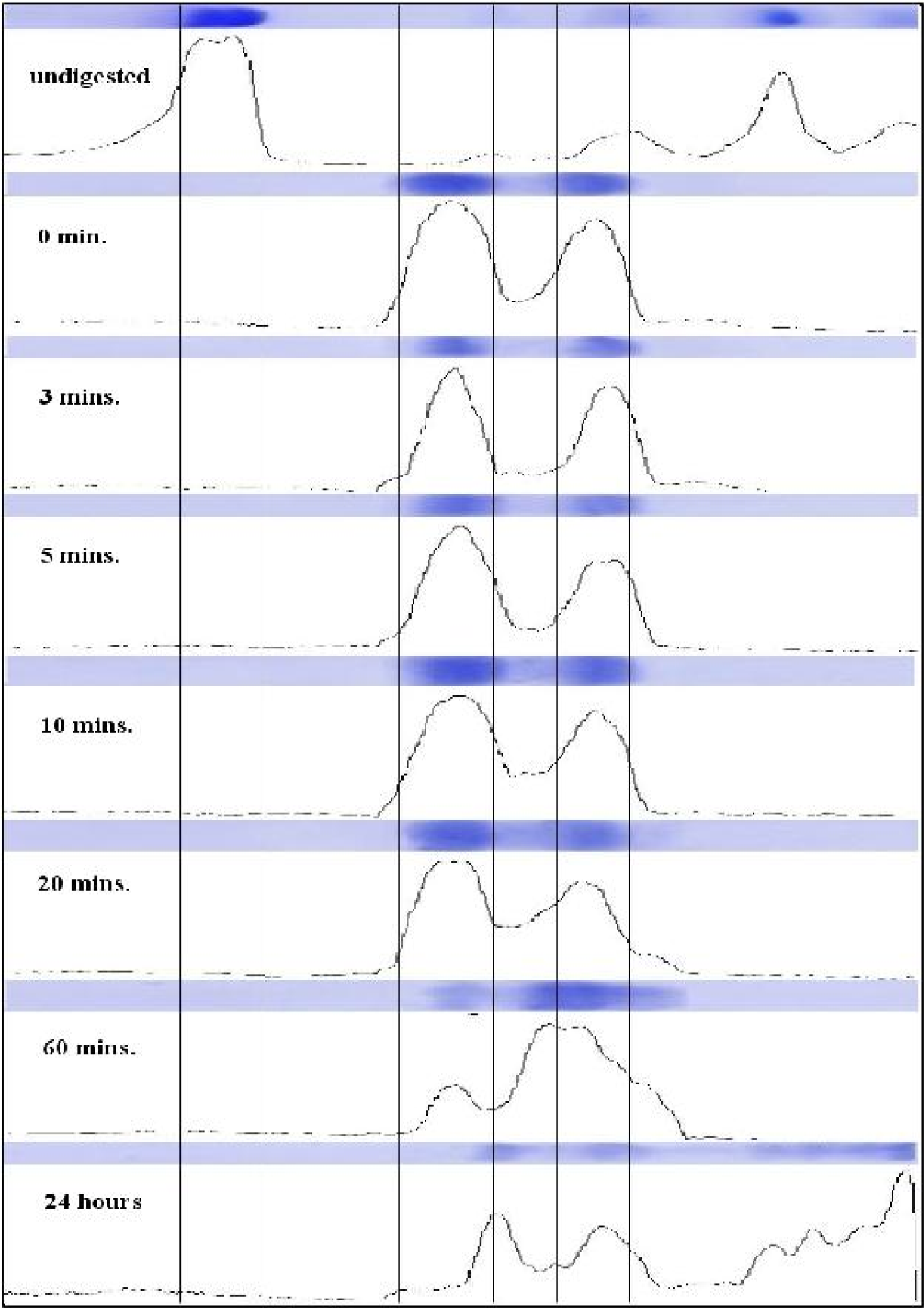


1. Pepsin-Trypsin


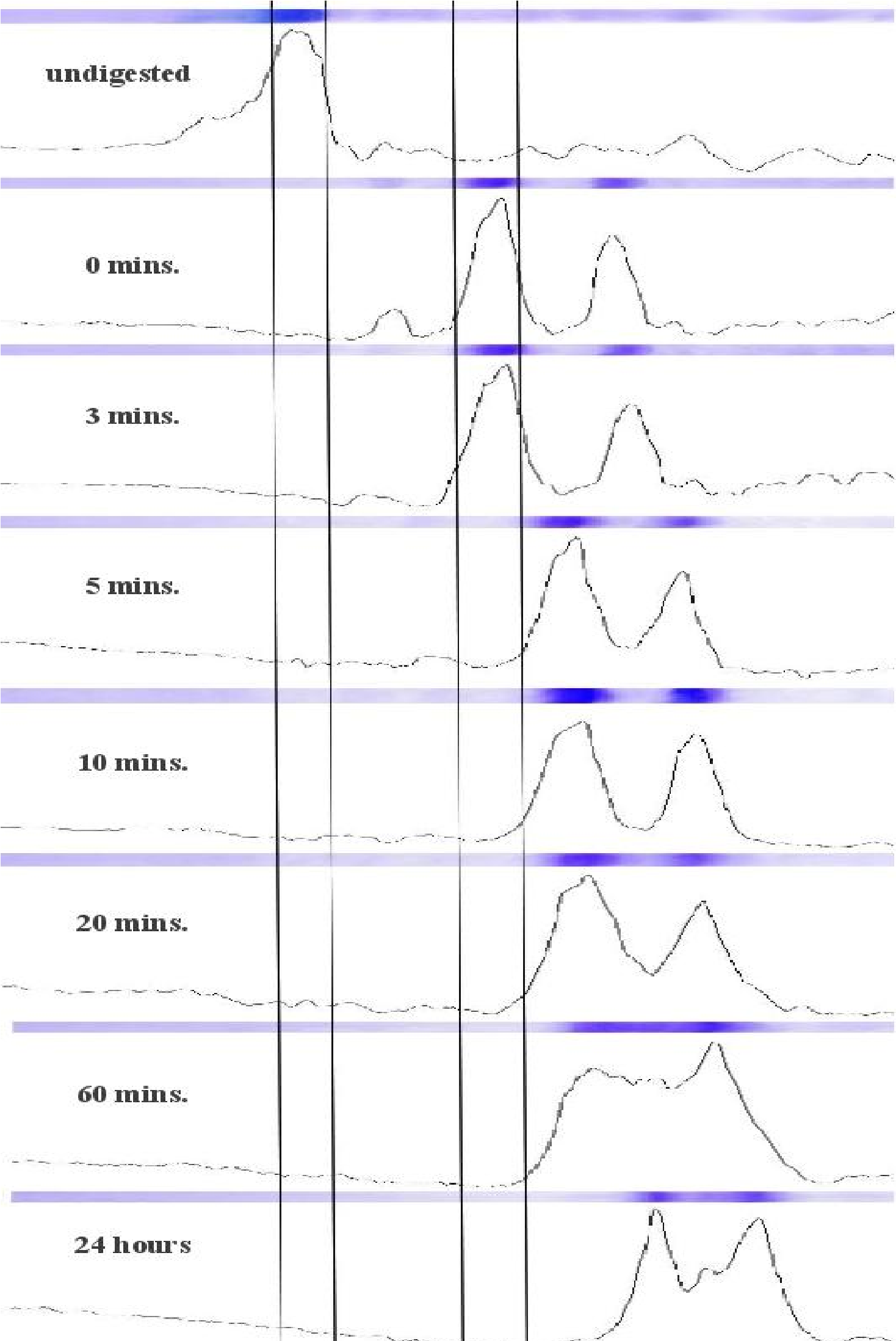


(E)Chymotrypsin-Trypsin


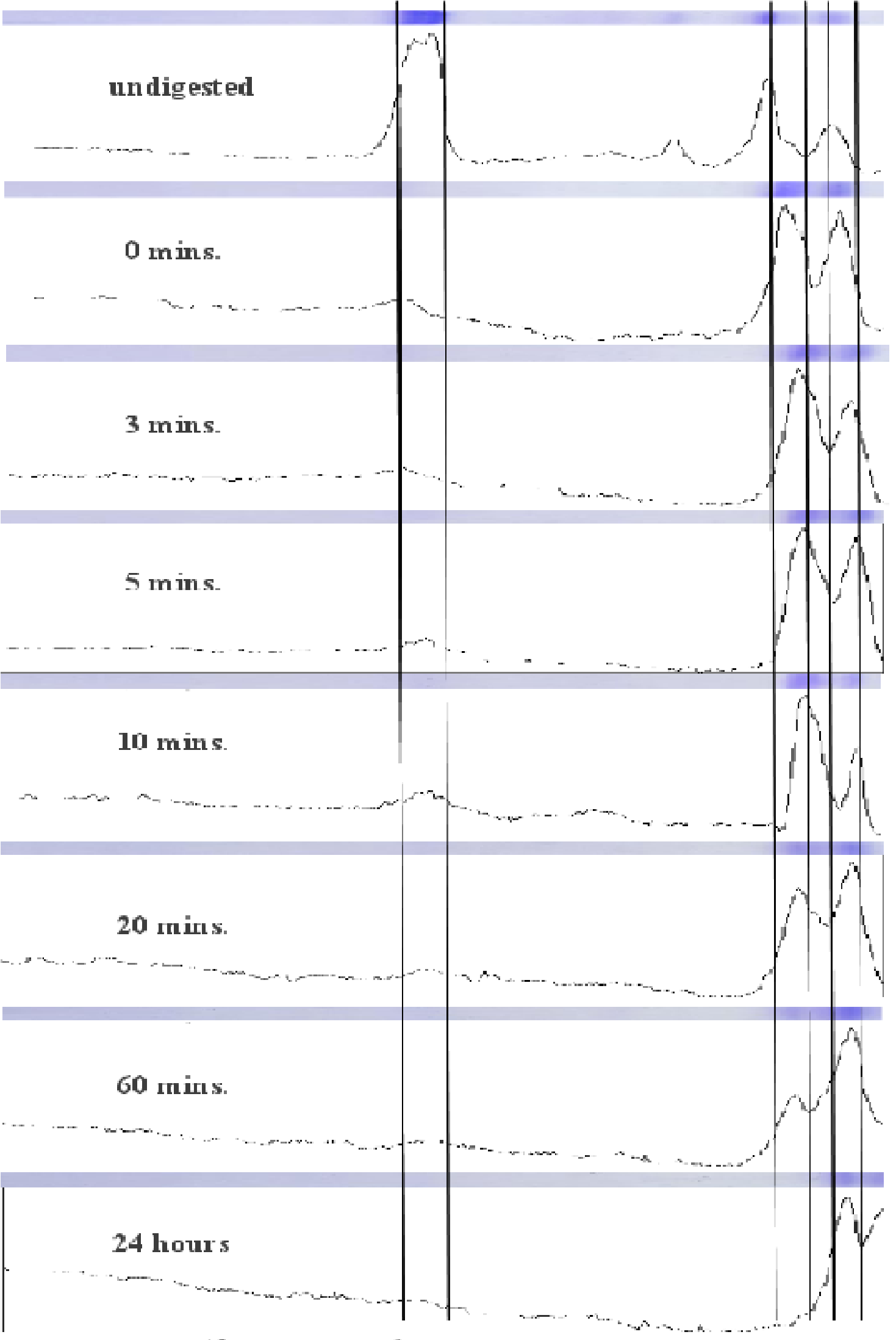


(F) Pepsin-Chymotrypsin-Trypsin

Appendix 3. Densitometric analysis of the protein digests using different enzymes (a-f: Pepsin,

Chymotrypsin, Trypsin, Pepsin-Trypsin, Chymotrypsin-Trypsin and Pepsin-ChymotrypsinTrypsin, respectively.)


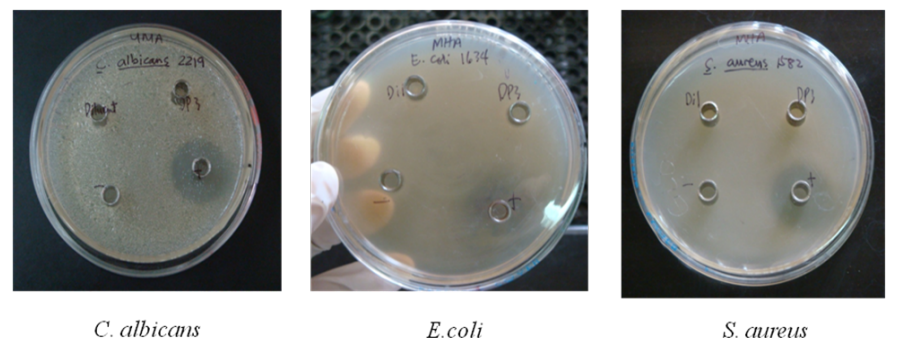


Appendix 4. Well-diffusion assay for the determination of antimicrobial activities of the protein digest using three different microbial strains (left to right: C. albicans 2219 on CMA, E.coli 1634 on MHA, and S. aureus 1582 on MHA). The test sample was labeled DP3, showing no zone of inhibition against each of the strains of microbes used. The positive control used (10% phenol) showed significant inhibitory activity across all microbial strains used.

Appendix 5. Statistical Analyses done in comparing the H_2_O_2_-scavenging activity of PCT digest and fractions.

# Results

# One-Way ANOVA

| One-Way ANOVA | | | | | | | | | | | |
| --- | --- | --- | --- | --- | --- | --- | --- | --- | --- | --- | --- |
|  |  |  |  |  |  |  |  |  |  |  |  |
|  | |  | | **F** | | **df1** | | **df2** | | **p** | |
| %scavenging activity |  | Welch's |  | 10.9 |  | 3 |  | 3.86 |  | 0.023 |  |
|  | | Fisher's |  | 10.1 |  | 3 |  | 8 |  | 0.004 |  |
|  | | | | | | | | | | | |

| Group Descriptives | | | | | | | | | | | |
| --- | --- | --- | --- | --- | --- | --- | --- | --- | --- | --- | --- |
|  |  |  |  |  |  |  |  |  |  |  |  |
|  | | **Samples** | | **N** | | **Mean** | | **SD** | | **SE** | |
| % Scavenging activity |  | Ascorbic acid |  | 3 |  | 3.16 |  | 0.7879 |  | 0.4549 |  |
|  |  | HPLC fraction 1 |  | 3 |  | 1.47 |  | 0.5474 |  | 0.3161 |  |
|  |  | HPLC fraction 2 |  | 3 |  | 1.51 |  | 0.0436 |  | 0.0252 |  |
|  |  | Protein Digest |  | 3 |  | 1.25 |  | 0.0700 |  | 0.0404 |  |
|  | | | | | | | | | | | |

## Assumption Checks

| Normality Test (Shapiro-Wilk) | | | | | |
| --- | --- | --- | --- | --- | --- |
|  |  |  |  |  |  |
|  | | **W** | | **p** | |
| %scavenging activity |  | 0.924 |  | 0.324 |  |
| Note. A low p-value suggests a violation of the assumption of normality | | | | | |
|  | | | | | |

| Homogeneity of Variances Test (Levene's) | | | | | | | | | |
| --- | --- | --- | --- | --- | --- | --- | --- | --- | --- |
|  |  |  |  |  |  |  |  |  |  |
|  | | **F** | | **df1** | | **df2** | | **p** | |
| %scavenging activity |  | 6.43 |  | 3 |  | 8 |  | 0.016 |  |
|  | | | | | | | | | |

## Post Hoc Tests

| Games-Howell Post-Hoc Test – %scavenging activity | | | | | | | | | | | |
| --- | --- | --- | --- | --- | --- | --- | --- | --- | --- | --- | --- |
|  |  |  |  |  |  |  |  |  |  |  |  |
|  | |  | | **Ascorbic acid** | | **HPLC fraction 1** | | **HPLC fraction 2** | | **Protein Digest** | |
| Ascorbic acid |  | Mean difference |  | — |  | 1.69 |  | 1.6500 |  | 1.910 |  |
|  |  | p-value |  | — |  | 0.132 |  | 0.163 |  | 0.125 |  |
| HPLC fraction 1 |  | Mean difference |  |  |  | — |  | -0.0400 |  | 0.220 |  |
|  |  | p-value |  |  |  | — |  | 0.999 |  | 0.894 |  |
| HPLC fraction 2 |  | Mean difference |  |  |  |  |  | — |  | 0.260 |  |
|  |  | p-value |  |  |  |  |  | — |  | 0.028 |  |
| Protein Digest |  | Mean difference |  |  |  |  |  |  |  | — |  |
|  |  | p-value |  |  |  |  |  |  |  | — |  |
|  | | | | | | | | | | | |

# References

**[1]** The jamovi project (2021). jamovi. (Version 1.6) [Computer Software]. Retrieved from <https://www.jamovi.org>.

**[2]** R Core Team (2020). R: A Language and environment for statistical computing. (Version 4.0) [Computer software]. Retrieved from <https://cran.r-project.org>. (R packages retrieved from MRAN snapshot 2020-08-24).

**[3]** Fox, J., & Weisberg, S. (2020). car: Companion to Applied Regression. [R package]. Retrieved from <https://cran.r-project.org/package=car>.

Appendix 6. Statistical Analyses done in comparing the Total scavenging activity of PCT digest and HPLC fractions.

# Results

# One-Way ANOVA

| One-Way ANOVA | | | | | | | | | | | |
| --- | --- | --- | --- | --- | --- | --- | --- | --- | --- | --- | --- |
|  |  |  |  |  |  |  |  |  |  |  |  |
|  | |  | | **F** | | **df1** | | **df2** | | **p** | |
| Total scavenging activity |  | Welch's |  | 78.5 |  | 2 |  | 3.03 |  | 0.002 |  |
|  | | Fisher's |  | 10.1 |  | 2 |  | 6 |  | 0.012 |  |
|  | | | | | | | | | | | |

| Group Descriptives | | | | | | | | | | | |
| --- | --- | --- | --- | --- | --- | --- | --- | --- | --- | --- | --- |
|  |  |  |  |  |  |  |  |  |  |  |  |
|  | | **Sample** | | **N** | | **Mean** | | **SD** | | **SE** | |
| Total scavenging activity |  | HPLC fraction 1 |  | 3 |  | 9.07e-4 |  | 6.66e-5 |  | 3.84e-5 |  |
|  |  | HPLC fraction 2 |  | 3 |  | 0.00110 |  | 3.61e-4 |  | 2.08e-4 |  |
|  |  | PCT digest |  | 3 |  | 3.50e-4 |  | 2.65e-5 |  | 1.53e-5 |  |
|  | | | | | | | | | | | |

## Assumption Checks

| Normality Test (Shapiro-Wilk) | | | | | |
| --- | --- | --- | --- | --- | --- |
|  |  |  |  |  |  |
|  | | **W** | | **p** | |
| Total scavenging activity |  | 0.879 |  | 0.151 |  |
| Note. A low p-value suggests a violation of the assumption of normality | | | | | |
|  | | | | | |

| Homogeneity of Variances Test (Levene's) | | | | | | | | | |
| --- | --- | --- | --- | --- | --- | --- | --- | --- | --- |
|  |  |  |  |  |  |  |  |  |  |
|  | | **F** | | **df1** | | **df2** | | **p** | |
| Total scavenging activity |  | 6.74 |  | 2 |  | 6 |  | 0.029 |  |
|  | | | | | | | | | |

## Post Hoc Tests

| Games-Howell Post-Hoc Test – Total scavenging activity | | | | | | | | | |
| --- | --- | --- | --- | --- | --- | --- | --- | --- | --- |
|  |  |  |  |  |  |  |  |  |  |
|  | |  | | **HPLC fraction 1** | | **HPLC fraction 2** | | **PCT digest** | |
| HPLC fraction 1 |  | Mean difference |  | — |  | -1.93e−4 |  | 5.57e-4 |  |
|  |  | p-value |  | — |  | 0.683 |  | 0.003 |  |
| HPLC fraction 2 |  | Mean difference |  |  |  | — |  | 7.50e-4 |  |
|  |  | p-value |  |  |  | — |  | 0.122 |  |
| PCT digest |  | Mean difference |  |  |  |  |  | — |  |
|  |  | p-value |  |  |  |  |  | — |  |
|  | | | | | | | | | |

Appendix 7. Statistical analysis on the comparisons on the % ACE-inhibition for each type of protein digest.

# Results

# One-Way ANOVA

| One-Way ANOVA | | | | | | | | | | | |
| --- | --- | --- | --- | --- | --- | --- | --- | --- | --- | --- | --- |
|  |  |  |  |  |  |  |  |  |  |  |  |
|  | |  | | **F** | | **df1** | | **df2** | | **p** | |
| %ACE inhibition |  | Welch's |  | 872 |  | 5 |  | 5.42 |  | < .001 |  |
|  | | Fisher's |  | 125 |  | 5 |  | 12 |  | < .001 |  |
|  | | | | | | | | | | | |

| Group Descriptives | | | | | | | | | | | |
| --- | --- | --- | --- | --- | --- | --- | --- | --- | --- | --- | --- |
|  |  |  |  |  |  |  |  |  |  |  |  |
|  | | **Protein Digest** | | **N** | | **Mean** | | **SD** | | **SE** | |
| %ACE inhibition |  | Chymotrypsin |  | 3 |  | 65.2 |  | 2.871 |  | 1.658 |  |
|  |  | Chymotrypsin-Trypsin |  | 3 |  | 36.6 |  | 0.700 |  | 0.404 |  |
|  |  | Pepsin |  | 3 |  | 60.7 |  | 1.982 |  | 1.144 |  |
|  |  | Pepsin-Chymotrypsin-Trypsin |  | 3 |  | 87.5 |  | 0.889 |  | 0.513 |  |
|  |  | Pepsin-Trypsin |  | 3 |  | 68.3 |  | 0.963 |  | 0.556 |  |
|  |  | Trypsin |  | 3 |  | 84.3 |  | 5.856 |  | 3.381 |  |
|  | | | | | | | | | | | |

## Assumption Checks

| Normality Test (Shapiro-Wilk) | | | | | |
| --- | --- | --- | --- | --- | --- |
|  |  |  |  |  |  |
|  | | **W** | | **p** | |
| %ACE inhibition |  | 0.920 |  | 0.127 |  |
| Note. A low p-value suggests a violation of the assumption of normality | | | | | |
|  | | | | | |

| Homogeneity of Variances Test (Levene's) | | | | | | | | | |
| --- | --- | --- | --- | --- | --- | --- | --- | --- | --- |
|  |  |  |  |  |  |  |  |  |  |
|  | | **F** | | **df1** | | **df2** | | **p** | |
| %ACE inhibition |  | 6.33 |  | 5 |  | 12 |  | 0.004 |  |
|  | | | | | | | | | |

## Post Hoc Tests

| Games-Howell Post-Hoc Test – %ACE inhibition | | | | | | | | | | | | | | | |
| --- | --- | --- | --- | --- | --- | --- | --- | --- | --- | --- | --- | --- | --- | --- | --- |
|  |  |  |  |  |  |  |  |  |  |  |  |  |  |  |  |
|  | |  | | **Chymotrypsin** | | **Chymotrypsin-Trypsin** | | **Pepsin** | | **Pepsin-Chymotrypsin-Trypsin** | | **Pepsin-Trypsin** | | **Trypsin** | |
| Chymotrypsin |  | Mean difference |  | — |  | 28.6 |  | 4.49 |  | -22.3 |  | -3.14 |  | -19.16 |  |
|  |  | p-value |  | — |  | 0.008 |  | 0.395 |  | 0.012 |  | 0.581 |  | 0.071 |  |
| Chymotrypsin-Trypsin |  | Mean difference |  |  |  | — |  | -24.07 |  | -50.9 |  | -31.70 |  | -47.72 |  |
|  |  | p-value |  |  |  | — |  | 0.004 |  | < .001 |  | < .001 |  | 0.016 |  |
| Pepsin |  | Mean difference |  |  |  |  |  | — |  | -26.8 |  | -7.63 |  | -23.65 |  |
|  |  | p-value |  |  |  |  |  | — |  | 0.002 |  | 0.047 |  | 0.052 |  |
| Pepsin-Chymotrypsin-Trypsin |  | Mean difference |  |  |  |  |  |  |  | — |  | 19.21 |  | 3.19 |  |
|  |  | p-value |  |  |  |  |  |  |  | — |  | < .001 |  | 0.908 |  |
| Pepsin-Trypsin |  | Mean difference |  |  |  |  |  |  |  |  |  | — |  | -16.02 |  |
|  |  | p-value |  |  |  |  |  |  |  |  |  | — |  | 0.137 |  |
| Trypsin |  | Mean difference |  |  |  |  |  |  |  |  |  |  |  | — |  |
|  |  | p-value |  |  |  |  |  |  |  |  |  |  |  | — |  |
|  | | | | | | | | | | | | | | | |

Appendix 8. Statistical analyses done on the comparison of IC_50_ values of the protein digests and the HPLC fractions.

# Results

# One-Way ANOVA

| One-Way ANOVA | | | | | | | | | | | |
| --- | --- | --- | --- | --- | --- | --- | --- | --- | --- | --- | --- |
|  |  |  |  |  |  |  |  |  |  |  |  |
|  | |  | | **F** | | **df1** | | **df2** | | **p** | |
| IC50, mg/ml |  | Welch's |  | 208 |  | 3 |  | 4.16 |  | < .001 |  |
|  | | Fisher's |  | 580 |  | 3 |  | 8 |  | < .001 |  |
|  | | | | | | | | | | | |

| Group Descriptives | | | | | | | | | | | |
| --- | --- | --- | --- | --- | --- | --- | --- | --- | --- | --- | --- |
|  |  |  |  |  |  |  |  |  |  |  |  |
|  | | **Sample** | | **N** | | **Mean** | | **SD** | | **SE** | |
| IC50, mg/ml |  | Captopril |  | 3 |  | 0.00379 |  | 8.19e-5 |  | 4.73e-5 |  |
|  |  | HPLC fraction 1 |  | 3 |  | 0.00535 |  | 1.64e-4 |  | 9.45e-5 |  |
|  |  | HPLC fraction 2 |  | 3 |  | 0.00432 |  | 9.26e-5 |  | 5.35e-5 |  |
|  |  | PCT digest |  | 3 |  | 0.03229 |  | 0.00199 |  | 0.00115 |  |
|  | | | | | | | | | | | |

## Assumption Checks

| Normality Test (Shapiro-Wilk) | | | | | |
| --- | --- | --- | --- | --- | --- |
|  |  |  |  |  |  |
|  | | **W** | | **p** | |
| IC50, mg/ml |  | 0.737 |  | 0.002 |  |
| Note. A low p-value suggests a violation of the assumption of normality | | | | | |
|  | | | | | |

| Homogeneity of Variances Test (Levene's) | | | | | | | | | |
| --- | --- | --- | --- | --- | --- | --- | --- | --- | --- |
|  |  |  |  |  |  |  |  |  |  |
|  | | **F** | | **df1** | | **df2** | | **p** | |
| IC50, mg/ml |  | 14.0 |  | 3 |  | 8 |  | 0.001 |  |
|  | | | | | | | | | |

## Post Hoc Tests

| Games-Howell Post-Hoc Test – IC50, mg/ml | | | | | | | | | | | |
| --- | --- | --- | --- | --- | --- | --- | --- | --- | --- | --- | --- |
|  |  |  |  |  |  |  |  |  |  |  |  |
|  | |  | | **Captopril** | | **HPLC fraction 1** | | **HPLC fraction 2** | | **PCT digest** | |
| Captopril |  | Mean difference |  | — |  | -0.00156 |  | -5.30e−4 |  | -0.0285 |  |
|  |  | p-value |  | — |  | 0.002 |  | 0.006 |  | 0.004 |  |
| HPLC fraction 1 |  | Mean difference |  |  |  | — |  | 0.00103 |  | -0.0269 |  |
|  |  | p-value |  |  |  | — |  | 0.006 |  | 0.004 |  |
| HPLC fraction 2 |  | Mean difference |  |  |  |  |  | — |  | -0.0280 |  |
|  |  | p-value |  |  |  |  |  | — |  | 0.004 |  |
| PCT digest |  | Mean difference |  |  |  |  |  |  |  | — |  |
|  |  | p-value |  |  |  |  |  |  |  | — |  |
|  | | | | | | | | | | | |
